# BCL-2 expression promotes immunosuppression in chronic lymphocytic leukemia by enhancing regulatory T cell differentiation and cytotoxic T cell exhaustion

**DOI:** 10.1186/s12943-022-01516-w

**Published:** 2022-02-22

**Authors:** Lu Liu, Xianfeng Cheng, Hui Yang, Senlin Lian, Yuegen Jiang, Jinhua Liang, Xiao Chen, Suo Mo, Yu Shi, Sishu Zhao, Jianyong Li, Runqiu Jiang, Dong-Hua Yang, Yujie Wu

**Affiliations:** 1 Department of Hematology, the First Affiliated Hospital of Nanjing Medical University, Jiangsu Province Hospital, Nanjing, 210029 China; 2 Key Laboratory of Hematology of Nanjing Medical University, Nanjing, 210029 China; 3 Department of Clinical laboratory, Institute of Dermatology, Chinese Academy of Medical Sciences and Peking Union Medical College, Nanjing, 210042 China; 4 Jiangsu Laboratory of Molecular Medicine, Medical School of Nanjing University, Nanjing, 210093 China; 5 State Key Laboratory of Pharmaceutical Biotechnology, Medical School, Nanjing University, Nanjing, 210093 China; 6 Department of Hepatobiliary Surgery, The Affiliated Drum Tower Hospital of Nanjing University Medical School, Nanjing, 210008 China; 7 Department of Pharmaceutical Sciences, College of Pharmacy and Health Sciences, St. John’s University, Queens, NY 11439 USA

**Keywords:** Chronic lymphocytic leukemia, T cells, BCL-2, Single-cell RNA sequencing

## Abstract

**Background:**

Chronic lymphocytic leukemia (CLL) results in increased susceptibility to infections. T cell dysfunction is not associated with CLL in all patients; therefore, it is important to identify CLL patients with T cell defects. The role of B-cell lymphoma-2 (BCL-2) in CLL has been explored; however, few studies have examined its role in T cells in CLL patients. Herein, we have investigated the regulatory role of BCL-2 in T cells in the CLL tumor microenvironment.

**Methods:**

The expression of BCL-2 in T cells was evaluated using flow cytometry. The regulatory roles of BCL-2 were investigated using single-cell RNA sequencing (scRNA-seq) and verified using multi-parameter flow cytometry on CD4 and CD8 T cells. The clinical features of BCL-2 expression in T cells in CLL were also explored.

**Results:**

We found a significant increase in BCL-2 expression in the T cells of CLL patients (*n* = 266). Single cell RNA sequencing (scRNA-seq) indicated that BCL-2^+^CD4^+^ T cells had the gene signature of increased regulatory T cells (Treg); BCL-2^+^CD8^+^ T cells showed the gene signature of exhausted cytotoxic T lymphocytes (CTL); and increased expression of BCL-2 was associated with T cell activation and cellular adhesion. The results from scRNA-seq were verified in peripheral T cells from 70 patients with CLL, wherein BCL-2^+^CD4^+^ T cells were enriched with Tregs and had higher expression of interleukin-10 and transforming growth factor-β than BCL-2^−^CD4^+^ T cells. BCL-2 expression in CD8^+^T cells was associated with exhausted cells (PD-1^+^Tim-3^+^) and weak expression of granzyme B and perforin. T cell–associated cytokine profiling revealed a negative association between BCL-2^+^ T cells and T cell activation. Decreased frequencies and recovery functions of BCL-2^+^T cells were observed in CLL patients in complete remission after treatment with venetoclax.

**Conclusion:**

BCL-2 expression in the T cells of CLL patients is associated with immunosuppression via promotion of Treg abundance and CTL exhaustion.

**Supplementary Information:**

The online version contains supplementary material available at 10.1186/s12943-022-01516-w.

## Background

Chronic lymphocytic leukemia (CLL) is a hematological malignancy characterized by the clonal proliferation of mature-appearing, small B lymphocytes in the peripheral blood, bone marrow, and lymphoid tissue [1]. CLL is the most prevalent adult leukemia in Western countries, representing approximately 34% of all new leukemia cases, and its incidence is increasing in China [2, 3]. The median age at diagnosis is 70 years, at which age, men are more likely to be affected than women (ratio of men to women, 1.7:1) [4]. A cardinal feature of the pathophysiology of CLL is immune system dysfunction, which is mainly manifested in humoral and cellular immunodeficiency status and a high incidence of autoimmune diseases, infections, and secondary malignancies [1, 5]. The use of cellular immunotherapy instead of chemotherapy has improved the treatment efficacy of CLL. However, despite the recent advances in treatment, CLL remains incurable [6–8].

Immune dysfunction is thought to be caused by an interaction between cancer cells and the immune system. These interactions provide a tumor microenvironment (TME) that enables the CLL cells to escape the immune surveillance machinery. TME is a heterogeneous, complex, and dominant tumor component. The importance of TME in the development and management of diseases has been demonstrated [9, 10]. Immune cells are a major component of the TME and are pivotal in the evaluation of prognoses and treatment responses of patients. T cells, such as regulatory T cells (Treg) and human naïve T cells, are involved in regulating tumorigenesis [11–13]. Understanding the immune cells and their cellular interactions in the TME is beneficial for the management of cancers. However, the regulatory roles of T cells within the TME in CLL remain unclear.

T cells play a central role in the establishment and maintenance of immune responses, memory, and homeostasis in antitumor immunity. They recognize diverse antigens from pathogens, tumors, and the environment, and maintain self-tolerance [14, 15]. They are grouped based on their functions into a series of subsets, such as naïve T cells that are able to respond to neoantigens, memory T cells that are derived from previous antigen activation and maintain long-term immunity, Tregs that can suppress immune responses, and others [16]. CLL is associated with profound defects in T cells and T cell functions, resulting in the failure of T cell antitumor activity and increased susceptibility to infections [17]. Not all CLL patients present T cell dysfunction; therefore, a marker to distinguish CLL patients with profound defects in T cell function is critical for the management of the disease [18].

B-cell lymphoma-2 (BCL-2) is the founding member of the BCL-2 family of proteins and plays an important role in promoting cellular survival via its anti-apoptotic function by inhibiting pro-apoptotic proteins. Dysregulation of the *BCL2* gene underlies many cancers, including melanoma, breast cancer, lung cancer, and chronic lymphocytic leukemia [19–21]. Although a significant increase in BCL-2 level is reported in T cells in patients with systemic lupus erythematosus, there are few studies on BCL-2 in T cells of patients with CLL [22]. Venetoclax, a BCL-2 inhibitor, has been proven to be highly effective in both CLL and acute myeloid leukemia (AML) and has been approved for the treatment of both diseases [23, 24]. It can increase T cell effector function by increasing reactive oxygen species (ROS) generation without inducing T cell apoptosis [25].

In the current study, we demonstrate that BCL-2 expression in T cells of CLL patients is associated with immunosuppressive TME by promotion of Treg abundance and cytotoxic T lymphocyte (CTL) exhaustion. Our data demonstrate that BCL-2 could be an important marker to distinguish patient populations with profound defects in T cell functions and highlight the possibility that BCL-2 inhibitors can restore T cell functions in patients with CLL.

## Methods

### Patients

This study was approved by the Ethics Committee of the Jiangsu Province Hospital (2018-SRFA-087), and written informed consent was obtained from all patients according to the Declaration of Helsinki. A total of 266 newly diagnosed patients with CLL in Jiangsu Province Hospital (Nanjing, China) from January 2017 to December 2021 and 30 age-matched healthy volunteers from our hematology laboratory were enrolled in this study (Table 1). Blood samples from six patients with CLL and two healthy donors were used for single-cell RNA sequencing (scRNA-seq) (Table 2). The functional verification experiments using flow cytometry (FCM) for BCL-2-positive T cells were performed in 70 CLL patients (Table 3). Finally, we collected 12 paired samples from patients who were in complete remission (CR) after venetoclax treatment. The diagnostic criteria were based on the International Workshop on CLL-National Cancer Institute (iwCLL-NCI) in 2018 [26], and the specimens included in the group were processed within 24 h of collection. The clinical characteristics of these patients with CLL are summarized in Tables 1, 2, 3.

**Table 1:** Clinical and biological characteristics of the 266 patients with CLL

characteristics		*N* = 266
Male, *n* (%)		167(62.8)
Median age (range), years		61(30–89)
Binet stage, n (%)	Stage A	69(25.9)
	Stage B/C	197(74.1)
Presence of B-symptoms, n (%)		51(19.2)
MBC > 5 × 10^9^/L, n (%)		210(78.9)
Platelets < 100 × 10^9^/L, n (%)		84(31.6)
Hemoglobin < 100 g/L, n (%)		58(21.8)
CD4/CD8	< 1, n (%)	52(19.5)
	> 2.5, n (%)	34(12.8)
β2-MG > 3.5 mg/L, n (%)		113(42.5)
Unmutated *IGHV*, n (%)		116(43.6)
Del(17p) or *TP53* mutation, n (%)		39(14.7)
Del(13q), n (%)		89(33.5)
Del(11q), n (%)		36(13.5)
Risk stratification, n (%)	low	68(25.6)
	medium	80(30)
	high	85(32)
	very high	33(12.4)

Abbreviations: *MBC* Monoclonal B cells, *β2-MG* β2-microglobulin, *IGHV* immunoglobulin heavy variable-region gene, *Del* delete, *TP53* tumor protein 53

**Table 2:** Clinical and biological characteristics of the patients with CLL whose samples were used for single-cell sequencing

Patients	CLL	CLL1	CLL2	CLL4	CLL5	CLL6	NC1	NC2
Sex	Male	Male	Female	Male	Male	Male	Male	Female
Age (years old)	65	54	68	60	58	45	66	49
Rai	IV	II	I	I	II	I	NA	NA
Binet	C	B	A	A	B	A	NA	NA
CLL-IPI risk	4	4	2	1	3	4	NA	NA
MBC (×10^9^/L)	25.5	64.3	16.9	5.84	57	54.8	NA	NA
Platelets (× 10^9^/L)	99	261	170	167	154	167	223	173
Hemoglobin (g/L)	123	158	119	146	129	146	158	142
CD4/CD8 (1–2.5)	1.8	0.5	4.7	NA	1.3	0.71	1.49	1.18
β2-MG (μmol/L)	2.85	1.9	2.37	NA	3.51	2.38	NA	NA
*IGHV* mutation	N	N	Y	Y	N	N	NA	NA
17p- / *TP53* mutation	Y	Y	N	N	N	Y	N	N

Abbreviations: *NA* not acquired, *MBC* Monoclonal B cells, *β2-MG* β2-microglobulin, *IGHV* immunoglobulin heavy variable-region gene, *N* no, *Y* yes, *17p-* 17p delete, *TP53* tumor protein 53

**Table 3:** Clinical and biological characteristics of the 70 CLL patients whose samples were used in the functional verification experiments

characteristics		*N* = 70
Male, n (%)		38(54.3)
Median age (range), years		57(30–87)
Binet stage, n (%)	Stage A	13(18.6)
	Stage B/C	57(81.4)
Presence of B-symptoms, n (%)		12(17.1)
MBC > 5 × 10^9^/L, n (%)		58(82.0)
Platelets < 100 × 10^9^/L, n (%)		23(32.9)
Hemoglobin < 100 g/L, n (%)		15(21.4)
CD4/CD8	< 1, n (%)	16(22.9)
	> 2.5, n (%)	9(12.9)
β2-MG > 3.5 mg/L, n (%)		25(35.7)
Unmutated *IGHV*, n (%)		28(40)
Del(17p) or *TP53* mutation, n (%)		10(14.3)
Del(13q), n (%)		23(32.9)
Del(11q), n (%)		6(8.6)
Risk stratification, n (%)	low	20(28.6)
	medium	20(28.6)
	high	20(28.6)
	very high	10(14.2)

Abbreviations: *MBC* Monoclonal B cells, *β2-MG* β2-microglobulin, *IGHV* immunoglobulin heavy variable-region gene, *Del* delete, *TP53* tumor protein 53

### Collection of single cells for scRNA-seq and flow cytometry

Peripheral blood mononuclear cells (PBMCs) were separated using density gradient centrifugation with Lymphocyte Separation Medium (LTS1077, TBD, Shanghai, China) solution as per the manufacturer’s instructions. In brief, 2 mL of fresh peripheral blood was collected from newly diagnosed CLL or healthy donors in anticoagulant tubes containing ethylenediaminetetraacetic acid (EDTA) and subsequently layered onto the separation medium. After centrifugation, the lymphocytic cells were carefully transferred to a new tube, washed twice with 1× phosphate-buffered saline (PBS; calcium-free and magnesium-free, HyClone, USA) containing 0.04% bovine serum albumin (BSA), and resuspended. The overall cell viability, which needed to be above 85%, was confirmed using the trypan blue exclusion method. The concentration of single cell suspensions was determined using a hemocytometer and adjusted to 700–1200 cells/μL.

### Chromium 10× genomics library and sequencing

Single cell suspensions were loaded onto a 10× chromium chip to capture 10,000 single cells—as much as possible—using a 10× Genomics Chromium Single-Cell 3′ reagent kit (V2) according to the manufacturer’s instructions (10× Genomics, CA). cDNA amplification and library construction were performed using reagents from the Chromium Single-Cell 3′ reagent kit (V2) according to the standard protocol. Libraries were sequenced on an Illumina NovaSeq 6000 sequencing system (paired-end multiplexing run, 150 bp) by LC-Bio Technology Co., Ltd. (Hangzhou, China) at a minimum depth of 20,000 reads per cell according to the manufacturer’s instructions (Illumina).

### Quality control and preprocessing of scRNA-seq data

The scRNA-seq raw data (BCL files) were converted into fastq files with bcl2fastq (Illumina) software. Reads were aligned to a human genome reference (GRCh38) and a digital gene expression matrix built using the STAR algorithm in CellRanger (10× Genomics; v3.0.2). Cells containing more than 200 expressed genes with a mitochondrial rate of less than 20% passed the cell quality filter, which was followed by the deletion of mitochondrial genes from the expression table. The Seurat package (version: 3.1.5, https://satijalab.org/seurat/) was utilized for cell normalization and regression based on the expression table according to the unique molecular identifier (UMI) counts of each sample and percentage of mitochondria to achieve scaled data. Each cell had 19,015 unique transcripts, and 41,618 cells were extracted from 44,291 cells through quality control, of which 2167 were T cells.

### Flow cytometry

**Fig. 1:**
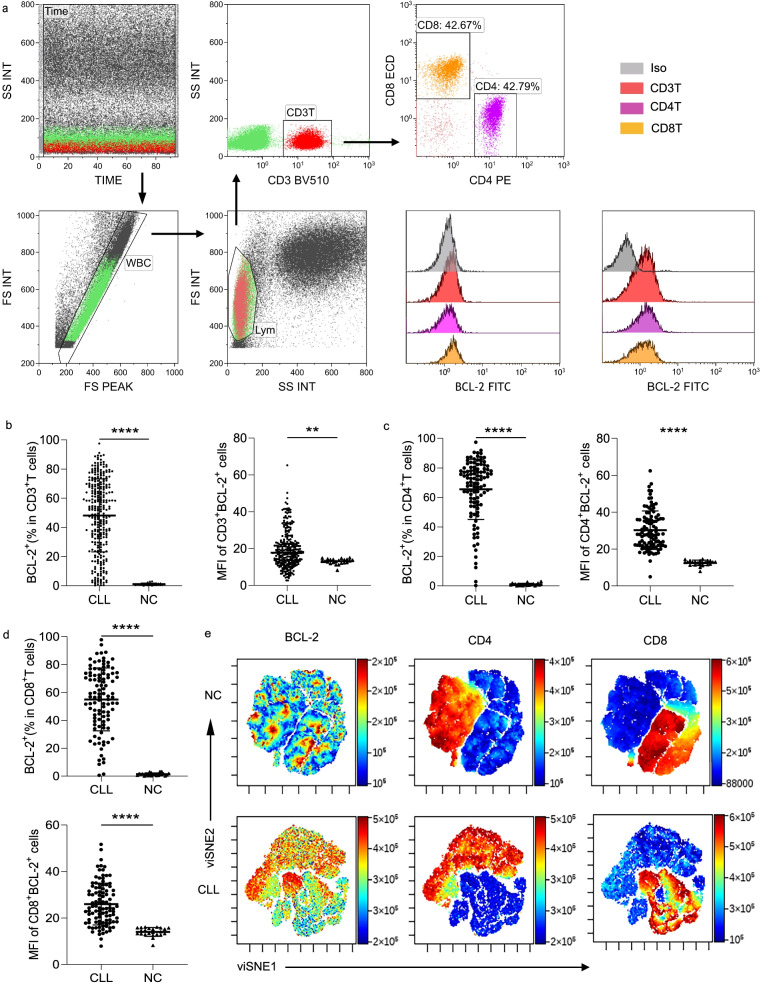
BCL-2 expression in chronic lymphocytic leukemia (CLL) and normal control (NC) T cells. (**a**) Flow cytometry gating strategy used for defining immune cell subsets. Representative examples of the difference in BCL-2 expression in CD3^+^ T, CD4^+^ T, and CD8^+^ T cells in NC and patients with CLL. (**b–d**) Percentages and MFI of cells positive for intracellular BCL-2 expression in CD3^+^ T (b, *n* = 266), CD4^+^ T (c, *n* = 110), and CD8^+^ T (d, *n* = 110) cells between NC and CLL. (**e**) Comparison charts generated by Cytobank on BCL-2 expression in 10 cases each of NC and CLL. Differences are shown using scatter plots (***p < 0.001, ****p < 0.0001)

Multi-parameter flow cytometry was used in all subsequent verification experiments, including experiments to determine the following: the differences in expression of programmed cell death protein 1 (PD-1), T cell immunoglobulin domain and mucin domain-3 (Tim-3), granzyme B, and perforin; the abundance of Tregs; the differentiation of T helper (Th) and memory T cells between BCL-2-positive and -negative T cells in CLL. Table S1 presents information about the antibodies and reagents used in these tests. Briefly, for determining the expression of PD-1, Tim-3, granzyme B, and perforin proteins, and the status of memory T cell differentiation, at least 2 × 10^6^ PBMCs were stained with antibodies against human CD3-BV510 (Clone UCHT1), CD4-PE (Clone SK3), CD8-ECD (Clone SFCI21Thy2D3), CD19-Percp-Cy5.5 (Clone SJ25C1), CD45RO-PE-Cy7 (Clone UCHL1), CD45RA-BV421 (Clone HI100), CD62L-APC/Cy7 (Clone DREG-56), PD-1-BV421 (Clone EH12.2H7), and Tim-3-APC (Clone F38-2E2) as per the manufacturers’ instructions for 15 min at 20 °C in the dark. For intracellular staining, surface-marked cells were fixed for 15 min and then permeabilized using an IntraStain Kit (Dako, DK) according to the manufacturer’s instructions after washing with 1× PBS (HyClone, USA) and centrifugation at 400×g for 5 min. Subsequently, the cells were stained with human BCL-2-FITC (Clone 124), granzyme B-PE (Clone GB11), and perforin-APC (Clone dG9) for 15 min and then washed once; the samples were kept on ice between sample processing and evaluation using FCM. Matched fluorescence mouse immunoglobulin (Ig)G1 was used as an isotype-matched control for important markers, including PD-1-BV421 (Clone EH12.2H7), Tim-3-APC (Clone F38-2E2), and FoxP3-PE (Clone PCH101). The stained samples were detected using Navios (Beckman Coulter, USA) and analyzed using Kaluza Analysis 2.1 (Beckman Coulter, USA). At least 200,000 cells were collected per tube according to the lymphocyte gate. First, single cells in the stable flow area were selected, and then lymphocyte populations were identified using side scatter (SSC) and forward scatter (FSC). Next, CD3 T, CD4 T, and CD8 T cells were identified using CD3-BV510 (Clone UCHT1), CD4-PE (Clone SK3), and CD8-ECD (Clone SFCI21Thy2D3) antibodies, respectively. Finally, the expression of various antigens mentioned above in each group was analyzed separately for BCL-2^+^ and BCL-2^−^ T cells (Fig. 1a).

### In vitro cell stimulation for cytokine detection

The differentiation of Th cells is defined by the expression of corresponding cytokines. To induce cytokine production, 2 × 10^6^ PBMCs were rested for 2 h and then stimulated with 2 μL Cell Activation Cocktail (with brefeldin A, BioLegend, USA) for 5 h at 37 °C in 1 mL complete medium in a 5% CO_2_ incubator. The complete medium contained RPMI 1640 medium (with 2 mM l-glutamine, Gibco, USA) supplemented with 10% heat-inactivated fetal bovine serum (Gibco, USA), 1% penicillin (100 U/mL, Gibco, USA), and streptomycin (100 μg/mL, Gibco, USA). Following stimulation, the expression of surface markers on the cells was evaluated by staining with antibodies, such as CD3-ECD (Clone UCHT1), CD4-PE (Clone SK3), CD4-PE-CY7 (Clone SK3), CD8-BV421 (Clone SK1), and CD185-APC (Clone J252D4), and then the cells were fixed and permeabilized using the IntraStain Kit, in accordance with the procedure described in the FCM section. Finally, the cells were stained for intracellular markers with BCL-2-FITC (Clone 124), interferon-gamma (IFN-γ)-PE (Clone 4S.B3), interleukin (IL)-4-BV510 (Clone MP4-25D2), IL-22-PE-Cy7 (Clone 2G12A41), and IL-17A-BV510 (Clone BL168) antibodies.

### Treg immunophenotype and related cytokine analyses

For Treg and related cytokine analyses, stimulated PBMCs were stained to evaluate the expression of markers with CD4-PE-CY7 (Clone SK3) and CD25-PC5 (Beckman Coulter) antibodies (15 min at 4 °C in the dark). After incubation, the cells were fixed, permeabilized, and stained using the eBioscience™ Human Regulatory T Cell Staining Kit (Invitrogen, USA) according to the manufacturer’s instructions. Finally, the cells were stained with intracellular antibodies including BCL-2-FITC (Clone 124), FoxP3-PE (Clone PCH101), IL-10-APC (Clone JES3-9D7), IL-35-AF700 (R&D Systems), and transforming growth factor (TGF)-β-BV421 (Clone TW7-16B4), in accordance with the previous approach outlined in the FCM section.

### β2-microglobulin (β2-MG) determination

Three milliliters of fasting venous blood were sampled from all subjects and centrifuged at 1610×g for 5 min to isolate the serum. Nephelometry was used to determine the β2-MG levels with an IMMAGE 800 Specific Protein Analyzer and the supporting kits (Beckman Coulter, USA). All experiments were performed in accordance with the manufacturer’s instructions.

### Statistical analysis

Data analysis was performed using IBM SPSS Statistics software (version 26.0, USA) for Windows. Measurement data are presented as mean ± standard deviation. Comparisons between two groups were analyzed using the Student’s *t-*test or Mann–Whitney U test for independent unpaired samples and paired Student’s *t-*test or Wilcoxon test for paired samples. The χ^2^ or Fisher’s exact test was used for analysis of categorical variables. All graphs were plotted using GraphPad Prism 9.0 (GraphPad Software, USA). For each FCM experiment, the data are representative of duplication of at least 45 biological samples. For the scRNA-seq experiment, the data are representative of the duplication of six biological samples. *P* < 0.05 was considered significant.

## Results

### BCL-2 expression increases in T cells and is associated with β2-MG concentration in CLL patients

To investigate the role of BCL-2 in T cells in CLL, we first investigated the expression of BCL-2 in T cells in patients with CLL (*n* = 266). The BCL-2 expression in T cells was gated as shown in Fig. 1a. Positive is defined as BCL-2 expression > 20%. We found that the BCL-2 level reflected by positive expression and mean fluorescence intensity (MFI) were all significantly increased in T cells of patients with CLL compared with those in healthy controls (Fig. 1b, S1a). Therefore, we investigated BCL-2 expression in CD4^+^ and CD8^+^ T cells in 110 patients with CLL and healthy controls using FCM. Both the protein expression and MFI of BCL-2 in CD4^+^ and CD8^+^ T cells were significantly higher in patients with CLL than in normal controls (NC) (Fig. 1c, d, S1b, c). Additionally, the FCM data from 10 NC or CLL cases were combined and analyzed using Cytobank to prepare a comparison chart of BCL-2 expression. The BCL-2 expression was higher in CLL (Fig. 1e). The correlation between the expression of BCL-2 in T cells and various clinical characteristics of patients with CLL is summarized in Table 4, which shows a positive correlation between BCL-2 expression in T cells and β2-MG (*χ*^*2*^ **=** 3.916, *P* < 0.05).

**Table 4:** Correlation between the level of BCL-2 in T cells and clinical characteristics of patients with CLL

characteristics		BCL-2 positive percentage		*χ*^*2*^	*P*
		< 20%	≥20%		
Sex	Male	27	140	1.516	0.218
	Female	22	77		
Age(years)	≤65	33	142	0.065	0.799
	> 65	16	75		
Binet stage	Stage A	14	55	0.263	0.877
	Stage B	18	80		
	Stage C	17	82		
β2-MG	≤3.5 mg/L	22	131	3.916	0.048
	> 3.5 mg/L	27	86		
*IGHV*	Mutated	28	122	0.014	0.906
	Unmutated	21	95		
*TP53* disruption	Yes	10	29	1.585	0.208
	No	39	188		
Del(13q)	Yes	16	73	0.018	0.895
	No	33	144		
Del(11q)	Yes	7	29	0.029	0.865
	No	42	188		
CLL-IPI risk	low	12	56	0.238	0.971
	medium	15	65		
	high	15	70		
	very high	7	26		

The tests used in Table 4 were all Chi-Square test or Fisher’s exact test

Abbreviations: *β2-MG* β2-microglobulin, *IGHV* immunoglobulin heavy variable-region gene, *TP53* tumor protein 53, *Del* delete, *IPI* International Prognostic Index

### BCL-2 expression in CD4^+^ T cells is related to activation and proliferation of Tregs

**Fig. 2:**
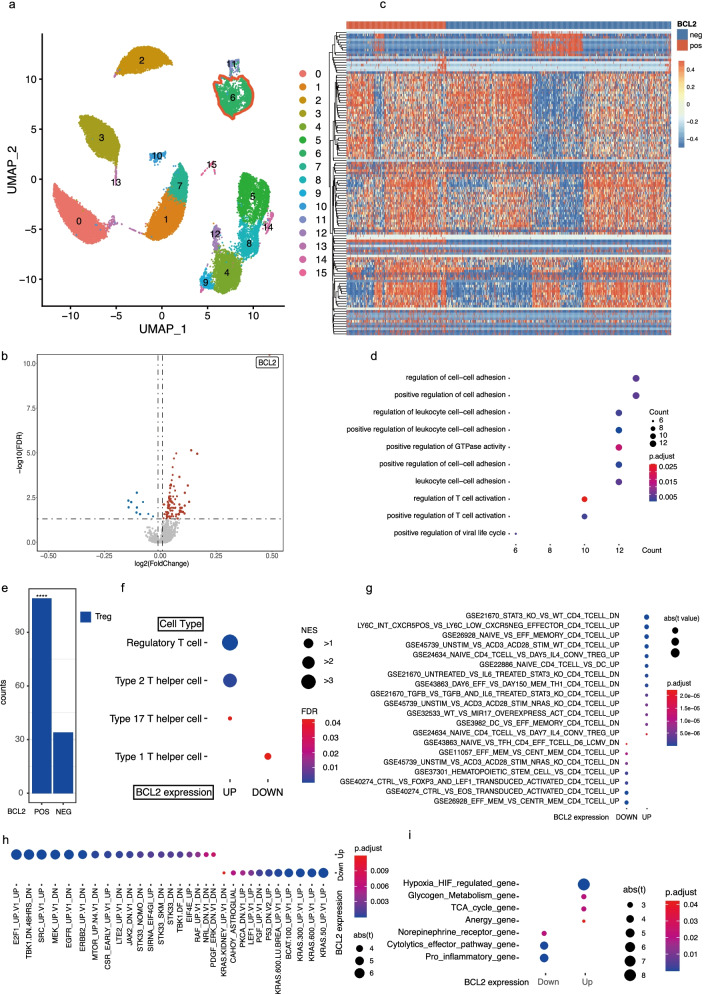
BCL-2 expression in CD4^+^ T cells is related to T cell activation, proliferation, and Tregs. (**a**) The Uniform Manifold Approximation and Projection (UMAP) plot of single cells obtained from six patients, representing 16 distinct clusters; cluster 6 stands for CD4^+^ T cells. Each dot corresponds to a single cell colored according to the cell clusters. (**b**) A volcano plot of differentially expressed genes (DEGs) to compare *BCL2*-positive and -negative CD4^+^ T cells. Each colored dot denotes an individual gene with an adjusted *p* value < 0.05. (**c**) Heatmap of DEGs compared between *BCL2*-positive and *BCL2*-negative CD4^+^ T cells. (**d**) GO enrichment analysis of DEGs. (**e**) Bar plot indicating Treg counts in *BCL2*-positive and -negative CD4^+^ T cells. (**f**) The dot plot of gene set enrichment analysis (GSEA) using helper T cell-related genes is indicated in the figure. (**g–i**) GSEA using IMMUNESIGDB, transcription factor target (TFT), and customized gene sets

To explore the detailed mechanism underlying BCL-2 expression in T cells in human CLL, we collected PBMC samples from six patients newly diagnosed with CLL (Table 2) and analyzed T cell subsets using scRNA-seq. Using Uniform Manifold Approximation and Projection (UMAP), we found that Cluster 6 indicated CD4^+^ T cells (Fig. 2a and Fig. S2). Gene expression in *BCL2*-positive and -negative cells was analyzed using the *limma* package, and the differentially expressed genes (DEGs) were plotted using a heatmap (Fig. 2b, c). Further enrichment analysis using KEGG indicated that the DEGs were mainly enriched in signaling pathways associated with “cell adhesion,” including “regulation of cell-cell adhesion,” “positive regulation of cell adhesion,” and “regulations of leukocyte cell-cell adhesion”. DEGs were also enriched in signaling pathways including “T cell activation” and “positive regulation of T cell activation” (Fig. 2d). Gene set enrichment analysis (GSEA) of the DEGs indicated that the *BCL2*-positive T cells had an increased number of Tregs and decreased expression of Th type 1 signature. Moreover, there were more Tregs in *BCL2*-positive T cells than in *BCL2*-negative T cells (Fig. 2e, f). We further confirmed the results of the gene set variation analysis (GSVA) using IMMUNESIGDB (ImmuneSigDB gene sets, 4872 gene sets). The results showed that *BCL2-* positive T cells have “IL-6 related signatures” and “T cell activation related signatures” (Fig. 2g). For the prediction of potential transcriptional factors (TFs), we used GSEA based on transcription factor target (TFT) gene sets (all TFTs, 1133 gene sets), and observed that *BCL2*-positive T cells exhibited signatures mainly involved in E2F transcription factor 1 and the epidermal growth factor receptor (EGFR)/MEK signaling pathway (Fig. 2h). Lastly, we analyzed *BCL2*-positive T cells for their metabolic features and found that *BCL2*-positive T cells were possibly related to “hypoxia inhibitory factor (HIF)_regulated gene” and “glycogen_metabolism_gene” (Fig. 2i).

### *BCL2* expression in CD8^+^T cells is related to increased exhaustion of cytotoxic T lymphocytes

**Fig. 3:**
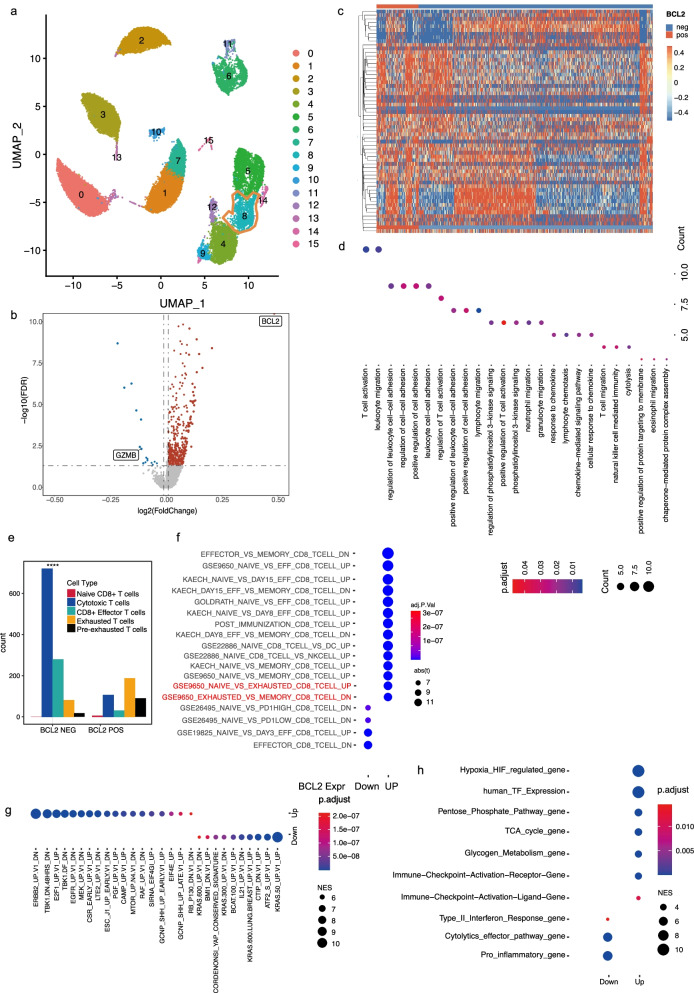
BCL-2 expression in CD8^+^ T cells is related to increased exhaustion of cytotoxic T lymphocytes. (**a**) The Uniform Manifold Approximation and Projection (UMAP) plot of single cells obtained from six patients, representing 16 distinct clusters; cluster 8 stands for CD8^+^ T cells. Each dot corresponds to a single cell colored according to the cell clusters. (**b**) A volcano plot of differentially expressed genes (DEGs) compared between *BCL2*-positive and -negative CD8^+^ T cells. Each colored dot denotes an individual gene with an adjusted *p* value < 0.05. (**c**) Heatmap of DEGs compared between *BCL2-*positive and *BCL2*-negative CD8^+^ T cells. (**d**) GO enrichment analysis of DEGs. (**e**) Bar plot of different linages of *BCL2*-positive and -negative CD8^+^ T cells. (**f**) Dot plot of gene set enrichment analysis (GSEA) enriched in lineages of CD8^+^ T cells. (**g**) and (**h**) GSEA using transcription factor target (TFT) and customized gene sets

We collected PBMC samples from six newly diagnosed CLL patients (Table 2) and analyzed CD8^+^T cells using scRNA-seq. We found that Cluster 8 represented CD8^+^T cells. We compared the gene expression between *BCL2*-positive and -negative CD8^+^T cells (Fig. 3a–c, and Fig. S2). Similar to the findings obtained using CD4^+^ cells, the KEGG enrichment analysis indicated that the DEGs are enriched in signaling pathways related to T cell activation and cell-cell adhesion and that *BCL2* expression in CD8^+^T cells is potentially related to cell migration, cytotoxic capacity, and activation of the PI3K AKT signaling pathway (Fig. 3d). The *BCL2* expression in CD8^+^T cells showed signatures of exhausted or malfunctioning T cells. First, we found that the counts of exhausted and pre-exhausted CD8^+^ T cells were significantly increased in *BCL2*-positive T cells. We further confirmed the results of the GSVA using IMMUNESIGDB (ImmuneSigDB gene sets, 4872 gene sets) and found that *BCL2*-positive CD8 cells have higher Normalized Enrichment Score (NES) in gene sets “GSE9650_NAIVE_VS_EXHAUSTED_CD8_TCELL_UP” and “GSE9650_NAIVE_VS_EXHAUSTED_CD8_TCELL_DOWN” than BCL2-negative cells. These results indicate that the transcriptional profile of *BCL2*-positive CD8 T cells was similar to that of exhausted T cells (Fig. 3e, f). In the TF prediction analysis, we found results similar to those of CD4^+^T cells, which indicate that both CD4^+^ and CD8^+^ T cells possibly share the same upstream signaling pathway (Fig. 3g). In the metabolic signature analysis, we found that HIF-regulated genes and pentose phosphate pathway genes were related to *BCL2*-positive CD8 cells (Fig. 3h). Furthermore, we compared the expression of cytolytic genes, including *GZMB*, *PRF1*, and *FSLG,* in *BCL2*-positive and -negative CD8^+^ T cells and found that *GZMB* and *PRF1* but not *FSLG* were expressed at significantly higher levels in *BCL2*-negative CD8^+^ T cells (Fig. S3a).

To find potential T cell *BCL2*-positive expression-related signaling pathways, we performed single-sample GSEA (ssGSEA) by using curated gene sets (C2, Ver. 7.4) in the Molecular Signatures Database (MSigDB) and further analysis of the correlation between *BCL2* expression in T cells and differentially expressed gene sets in MSigDB C2 by multiple correlation analysis. The results indicate that the gene sets including “BH3_ASSOCIATE_WITH_BCL_2_MEMBERS” and “BAKER_HEMATOPOESIS_STAT5_TARGETS” are not only differently expressed between *BCL2*-negative and -positive T cells but also well-correlated with *BCL2* expression in T cells (Fig. S3b, c).

### BCL-2 expression is related to decreased Th1 and increased Treg percentages in patients with CLL

**Fig. 4:**
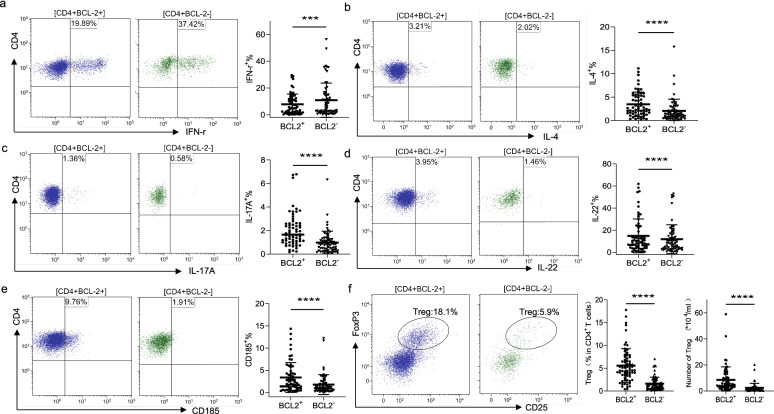
Distribution of different lineages of CD4^+^ T cells in BCL-2-positive and -negative CLL (*n* = 70). (**a–e**) Representative flow cytometry scatter plots of Th1 IFN-γ^+^, Th2 IL-4^+^, Th17 IL-17A^+^, Th22 IL-22^+^, and Tfh CD185^+^ cells in BCL-2^+^ and BCL-2^−^ CD4^+^ T cells in patients with CLL. Percentage of cells positive for each part expression in CD4^+^ T cells of BCL-2-positive and -negative T cells is shown. (**f**) Representative examples of Tregs in CD4^+^BCL-2^+^ T and CD4^+^BCL-2^−^ T cells in patients with CLL. Increased Treg frequency and absolute cell numbers in CD4^+^BCL-2^+^ T cells of patients with CLL are shown. Differences are shown in scatter plots (***p < 0.001, ****p < 0.0001)

To verify these findings, we collected PBMC samples from 70 patients newly diagnosed with CLL (Table 3). First, we focused on the impact of BCL-2 expression on the percentage of differentiated Th cells, including Th1, Th2, Th17, Th22, Tregs, and follicular helper T cells (Tfh). BCL-2^+^ Th cells had a significantly lower percentage of Th1 cells (BCL-2^+^ vs. BCL-2^−^: 7.7 ± 7.7% vs 10.9 ± 12.8%, *P* < 0.05) (Fig. 4a) and significantly higher percentage of Th2, Th17, Th22, and Tfh cells (BCL-2^+^ vs. BCL-2^−^: 3.5 ± 2.6% vs. 3.0 ± 6.5%, Th2; 2.0 ± 1.4% vs. 1.0 ± 1.0%, Th17; 14.8 ± 15.3% vs. 11.8 ± 13.0%, Th22; and 3.6 ± 4.1% vs. 1.8 ± 2.2%, Tfh, *P* < 0.05) (Fig. 4b–e). Notably, BCL-2 expression in T cells had the greatest impact on Tregs (CD4^+^CD25^high^FoxP3^+^ T cells) not only in percentage but also in absolute number. The percentage of Tregs (Tregs/total CD4^+^ cells) was significantly higher in BCL-2^+^ Th cells than in BCL-2^−^Th cells (BCL-2^+^ vs. BCL-2^−^: 5.5 ± 3.7% vs. 1.5 ± 1.8%, *P* < 0.01). The absolute number of Tregs per mL was 8.6 ± 10.0 × 10^4^ Tregs in the BCL-2^+^CD4^+^T cells and 2.4 ± 3.4 × 10^4^ Tregs in the BCL-2^−^CD4^+^T cells (Fig. 4f). We found that BCL-2 expression in T cells can affect the Th subsets.

### BCL-2 expression is associated with immunosuppression reflected by increasing IL-10 and TGF-β in Tregs

**Fig. 5:**
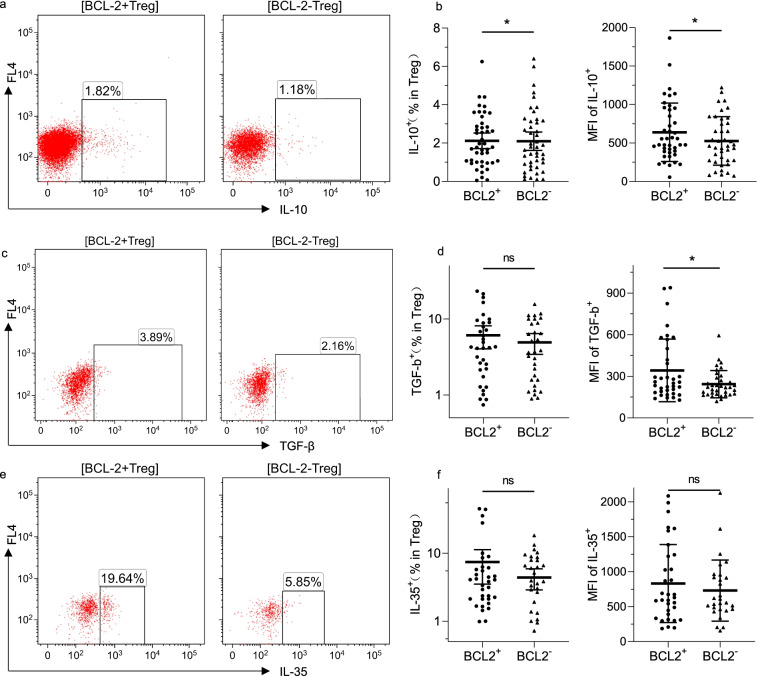
Cytokines secreted from Tregs in BCL-2-positive and -negative CD4^+^T cells in patients with CLL (*n* = 45). (**a**), (**c**), and (**e**) Representative examples of IL-10, TGF-β, and IL-35 in BCL-2^+^ and BCL-2^−^ Tregs. (**b**), (**d**), and (**f**) Percentage positive and MFI of IL-10, TGF-β, and IL-35 among Tregs between BCL-2-positive and -negative T cells. Differences are shown in scatter plots (*p < 0.05; ns, not significant)

BCL-2 was found to be related to increased Treg percentages in Th cells. We further analyzed whether BCL-2 has an impact on the production of Treg-derived suppressive cytokines in the PBMCs of 45 patients with CLL; these cytokines included IL-10, IL-35, and TGF-β. TGF-β is essential for the differentiation of naïve CD4^+^ cells into Tregs and is important for maintaining Treg homeostasis [27]. We found that the percentage of IL-10 and the MFI of IL-10 and TGF-β in the BCL-2^+^ Tregs increased significantly compared with the corresponding values in the BCL-2^−^ Tregs (Fig. 5a-d). However, the percentages of IL-35^+^ and TGF-β^+^ cells in the BCL-2^+^ Tregs were not statistically different compared with the percentages in the BCL-2^−^Tregs, whereas the BCL-2^+^ Tregs could secrete more IL-35 and TGF-β (Fig. 5d, f).

### BCL-2 promotes cell exhaustion and reduced cytotoxicity of CD8^+^T cells in patients with CLL

**Fig. 6:**
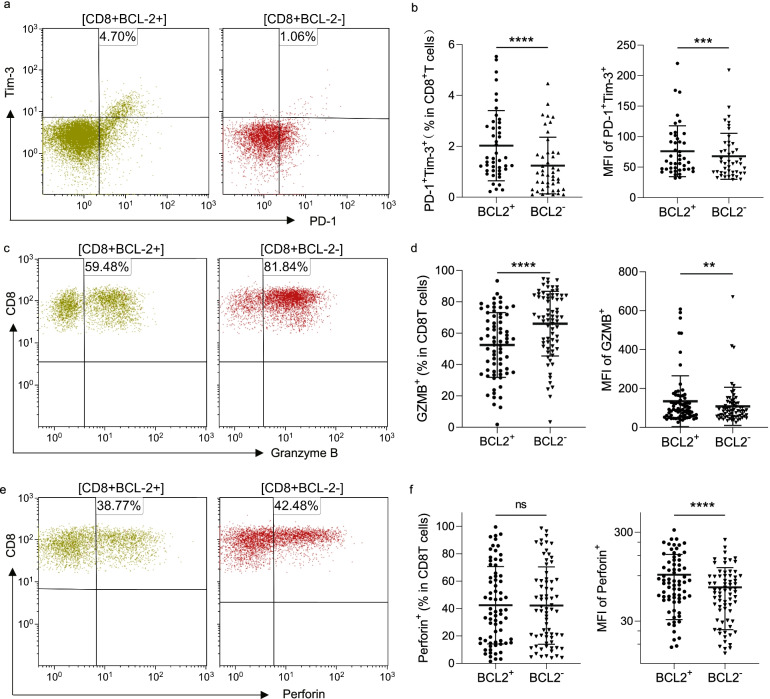
Exhaustion and cytotoxicity of CD8^+^ T cells (*n* = 70). (**a**) Representative graphs of PD1^+^Tim-3^+^ in BCL-2^+^, and BCL-2^−^ CD8^+^ T cells of patients with CLL. (**b**) Comparison of the percent positive rate and MFI of PD1^+^Tim-3^+^ in BCL-2^+^ and BCL-2^−^ CD8^+^ T cells. (**c**) and (**e**) Representative graphs of granzyme B and perforin in CD8^+^BCL-2^+^ T and CD8^+^BCL-2^−^ T cells in patients with CLL. (**d**) and (**f**) Percentage positive and MFI of granzyme B and perforin in BCL-2-positive and -negative T cells. Differences are shown in scatter plots (**p < 0.01, ***p < 0.001, ****p < 0.0001, ns, not significant)

We investigated the association between BCL-2 expression in CD8^+^ T cells and their exhaustion and cytotoxicity in 70 patients with CLL. We found that there was a significantly higher proportion and MFI of exhausted CD8^+^T cells (PD-1^+^Tim-3^+^CD8^+^) in the CD8^+^BCL-2^+^ T cells than in the CD8^+^BCL-2^−^ T cells (BCL-2^+^ vs. BCL-2^−^: 2.39 ± 2.23% vs. 1.44 ± 1.46% and 76.1 ± 41.8 vs. 62.7 ± 28.9% for proportion and MFI, respectively, *P* < 0.05) (Fig. 6a, b). In addition to causing T cell exhaustion, increased BCL-2 expression was associated with decreased expression of hallmarked proteins, including granzyme B and perforin. Furthermore, the percentage of granzyme B positivity in CD8^+^ BCL-2^+^ T cells decreased (BCL-2^+^ vs. BCL-2^−^: 52.5 ± 20.7% vs. 66.1 ± 20.7%, *P* < 0.05) while that of perforin in CD8^+^BCL-2^+^ T cells remained unchanged (Fig. 6c–f). However, unexpectedly, the MFI levels of granzyme B and perforin in the CD8^+^BCL-2^+^ T cells were higher than those in the CD8^+^BCL-2^−^T cells (Fig. 6c–f).

### BCL-2 alters T cell differentiation in patients with CLL

**Fig. 7:**
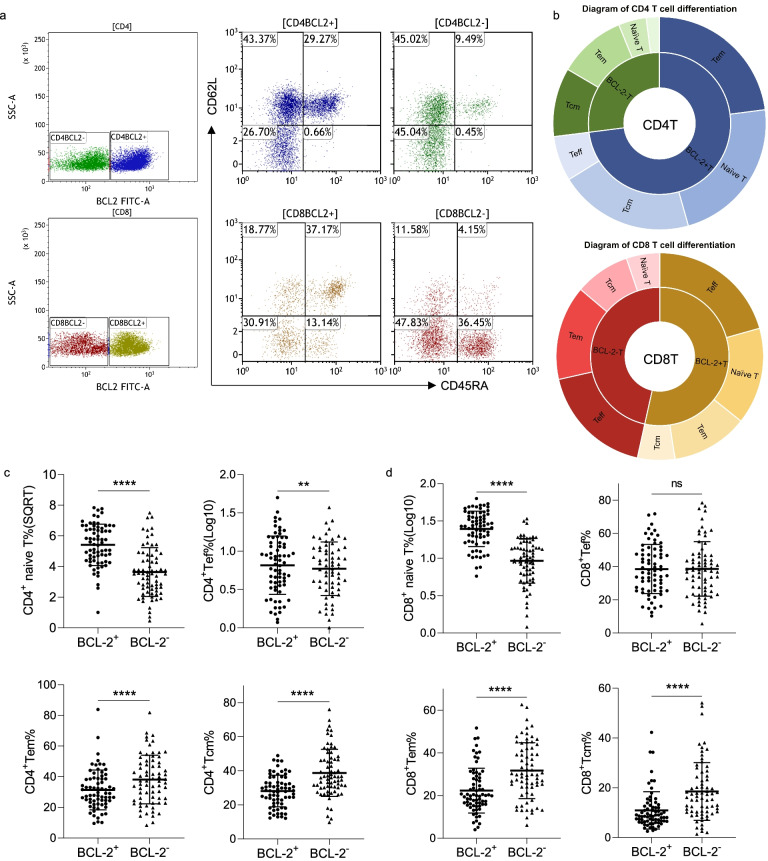
Differentiation of BCL-2-positive and -negative T cells in CLL (*n* = 70). (**a**) Representative examples of naïve T cells, effector T cells (Teff), effector memory T cells (Tem), and central memory T cells (Tcm) in CD4^+^ and CD8^+^ T cells in patients with CLL. (**b**) Sunburst diagram for differentiation of CD4^+^ and CD8^+^ T cells. (**c**) and (**d**) Percentage of cells positive for CD4^+^ and CD8^+^ T cells between BCL-2-positive and -negative T cells. Differences are shown in scatter plots (**p < 0.01, ****p < 0.0001, ns, not significant)

Generally, the differentiation of T cells can be divided into four stages based on the expression of CD45RA and CD62L, including naïve T cells (Tn, CD45RA^+^CD62L^+^), effector T cells (Teff, CD45RA^+^CD62L^−^), effector memory T cells (Tem, CD45RA^−^CD62L^−^), and central memory T cells (Tcm, CD45RA^−^CD62L^+^) (Fig. 7a, b). Seventy patients who were newly diagnosed with CLL were involved in this study. BCL-2 expression in each T cell subset was assessed, and the results indicate that the proportion of naïve T cells in the BCL-2^+^ T cells was higher than that in the BCL-2^−^ T cells in both the CD4^+^ (Fig. 7c) and CD8^+^ T cells (Fig. 7d). Moreover, the proportion of Tem and Tcm in the BCL-2^+^ T cells was lower than that in the BCL-2^−^ T cells. Additional File 4 shows the percentage of Tn, Tem, Tcm, and Teff without the BCL-2 subgrouping in the CD4^+^ and CD8^+^ T cells in the 70 patients with CLL (Fig. S4a, b).

### Decreased frequency and recovery function of BCL-2^+^ T cells in patients with CLL in CR

**Fig. 8:**
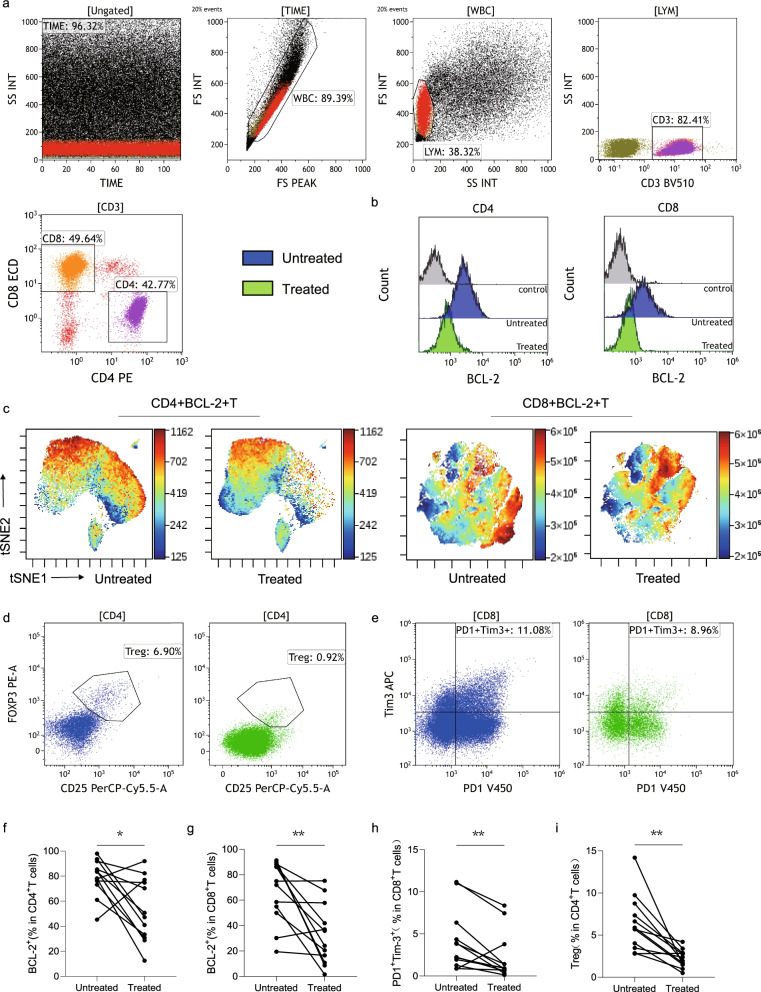
BCL-2 expression and restored function in T cells after venetoclax treatment in patients with chronic lymphocytic leukemia (CLL) (*n* = 12). (**a**) Flow cytometry gating strategy used for defining immune cell subsets. (**b**) Flow cytometry representative plots of BCL-2 expression in CD4^+^ and CD8^+^ T cells between untreated (blue) and treated (green) CLL patients. (**c**) The tSNE graphs show BCL-2 expression in CD4^+^ and CD8^+^ T cells between untreated and treated CLL patients. (**d**) and (**e**) Flow cytometry representative plots of Tregs in CD4^+^ T cells (**d**) and PD1^+^Tim-3^+^ in CD8^+^ T cells (**e**) pre- and post- venetoclax treatment. (**f**) and (**g**) Percentage BCL-2 positivity in CD4^+^ (**f**) and CD8^+^ (**g**) T cells between untreated and treated patients with CLL. (**h**) and (**i**) Percentage of Tregs in CD4^+^ T cells (**h**) and PD1^+^Tim-3^+^ in CD8^+^ T cells (**i**) pre- and post- venetoclax treatment. Differences are shown as before-after plots (*p < 0.05, **p < 0.01)

Finally, we compared the BCL-2 expression and function of the peripheral T cells derived from 12 matched patients with CLL pre- and post-venetoclax treatment (Fig. 8a–e). We found that venetoclax significantly decreased the percentage of CD4^+^BCL-2^+^ and CD8^+^BCL-2^+^ T cells in patients with CLL (Fig. 8f, g). Furthermore, the percentage of Tregs in CD4^+^ T cells and PD1^+^Tim-3^+^ cells in CD8^+^ T cells decreased significantly with venetoclax treatment (Fig. 8h, i). These results indicate that BCL-2 expression in peripheral T cells is a potential indicator of the therapeutic response.

## Discussion

CLL is a mature B-cell malignancy, which is closely associated with profound alterations and defects in the immune system, particularly with respect to the complex relationship between T cells and leukemia cells. Information regarding T cell characteristics in CLL has grown steadily in recent years, but the answer to the question of whether T cells act as bystander cells or possess antitumor activity is still unclear. In this study, we investigated the function of BCL-2-positive T cells in CLL using FCM. We found that there were BCL-2-positive and -negative T cells in patients with CLL. The differences in gene expression between BCL-2-positive and -negative T cells in CLL were validated by single-cell sequencing. The parallel use of single-cell sequencing approaches and FCM decreased the chances of bias. We demonstrated that the BCL-2-positive T cells of patients with CLL exhibit profound defects in T cell functions and T cell differentiation.

Overexpression of BCL-2 has been extensively reported in patients with CLL, and the possible mechanisms underlying this phenomenon include hypomethylation of the BCL-2 gene and deletion of the genes encoding miR-15 and miR-16 at 13q14. Importantly, related inhibitors that downregulate BCL-2 expression in immune cells have been developed and approved for CLL treatment. Venetoclax is an approved chemotherapy drug for CLL, which inhibits BCL-2 expression in tumor cells as well as in immune T lymphocytes [28–32]. However, there are only a few reports on the expression of BCL-2 in T cells in CLL. Therefore, we investigated the role of BCL-2 in T cells from patients with CLL. We found that BCL-2 was overexpressed in patients with CLL and that this overexpression was associated with immunosuppression of CLL, with an increased percentage of Tregs and exhausted CTLs. We noticed a positive correlation between BCL-2 expression in T cells and β2-MG levels. Serum β2-MG concentration is an independent prognostic factor according to the CLL-International Prognostic Index (CLL-IPI), i.e., the higher the β2-MG level, the worse the prognosis [26]. This suggested that we could use BCL-2 to assist the prognostic stratification of CLL patients. Additionally, we explored the relationship between BCL-2 expression and other factors. Many studies have shown that BCL-2 exerts its tumor-promoting function through cooperation with anti-apoptotic proteins and inhibition of pro-apoptotic proteins [33]. Consistent with our results, BCL-2 expression has been associated with other BCL-2 family members and the STAT5 pathway. For example, it has been reported that STAT5 can activate the expression of key proto-oncogenes such as BCL-2 [34, 35].

Increased Tregs in the TME are one of the reasons for immune escape in CLL [36, 37]. Tregs promote immunosuppression by releasing IL-10 and TGF-β [6, 38–42]. Weiss et al. [43] found that high Treg levels are an indicator for predicting the time to initial treatment in patients with CLL in the low to intermediate stages. In the present study, we found a significantly higher proportion and quantity of Tregs in CD4^+^BCL-2^+^ T cells than in CD4^+^BCL-2^−^T cells. The proportion and MFI of IL-10 secreted from BCL-2^+^ Tregs were significantly increased compared with those secreted from BCL-2^−^ Tregs. Therefore, we postulated that CLL patients with high BCL-2 expression in T cells might have a poorer prognosis than CLL patients with low BCL-2 expression in T cells due to immunosuppression. Our hypothesis was supported by a recent publication, which indicated that BCL-2 is critical for antitumor immune responses [44].

T cell exhaustion is a unique phenotype of T cells as a result of long-term exposure to antigens in the presence of inflammatory cytokines, which is characterized by loss of effector function, poor proliferation, and reduced cytotoxicity [45, 46]. Inhibitory receptors, such as PD-1 and Tim3, play an essential role in the regulation of T cell response and are closely associated with T cell exhaustion [47, 48]. T cell dysfunction in CLL occurs through direct and indirect interactions of CLL cells with both CD4^+^ and CD8^+^ T cells. The high levels of inhibitory molecules of PD-L1, CD200, B7-H3 and CD27 are key mediators of acquired T cell defects through binding to PD-1, CD200R, and B- and T lymphocyte attenuator (BTLA) on T cells [17, 49]. PD-1 blockade has achieved the highest response rate in classical Hodgkin lymphoma and has also shown clinical activity in several types of B-cell non-Hodgkin lymphoma with variable PD-L1 expression, especially in CLL patients with Richter transformation [50, 51]. Our study provides a new explanation for the increased T cell exhaustion in patients with CLL. We found that CD8^+^BCL-2^+^ T cells displayed a significantly higher proportion and MFI of PD-1^+^Tim-3^+^ cells than CD8^+^BCL-2^−^T cells in patients with CLL. The percentage of granzyme B positivity in CD8^+^BCL-2^+^ T cells was also lower than that in CD8^+^BCL-2^−^ T cells. Perforin and granzymes released by CTLs have been shown to synergistically mediate the apoptosis of target cells, and a decrease in granzyme B indicates a decrease in T cell cytotoxicity [52–54]. Our results indicated that CD8^+^BCL-2^+^ T cells have an exhausted phenotype and weakened cytotoxic functions, thereby indicating that BCL-2 overexpression is associated with T cell exhaustion. With respect to the increased MFI of granzyme B and perforin in CD8^+^BCL-2^+^ T cells, we speculated that this phenomenon could compensate for the insufficient amount of granzyme B and perforin secreted by the CD8^+^BCL-2^+^ T cells. This observation suggested that the response of T cells to chronic T cell receptor (TCR) stimulation gradually enters a dysfunctional state after the initial differentiation and expansion of CD8^+^ effector cells [55].

The four stages of T cell differentiation, including naïve T-cells, Teff, Tem, and Tcm, are important to understand immune responses and immune profiling. Naïve T cells quickly differentiate into Teff to help our body resist new, unrecognized infections and diseases, while Tem are enriched in response to recalled antigens. Specifically, Teff respond to active antigenic stimulation to eliminate pathogens [56, 57]. Patients with CLL display a subset distribution skewed toward an effector memory phenotype, particularly in cytomegalovirus-positive patients [17, 58–61]. In our study, we found an adverse differentiation of T cells between high and low BCL-2 expression in patients with CLL, and most of the differentiation of BCL-2-positive T cells was blocked in the naïve T cell stage. Similar results were observed in both CD4^+^ and CD8^+^ T cells. The proportion of Tem and Tcm in BCL-2^+^ T cells was lower than that in BCL-2^−^ T cells. These results were statistically significant and demonstrated that the ability of BCL-2^+^ T cells to differentiate into effector T cells was compromised.

Low rates of progression and high rates of minimal residual disease negativity are observed with the combination therapy of venetoclax and anti-CD20 monoclonal antibodies, such as obinutuzumab [62, 63]. However, the effect of BCL-2 inhibitors on non-malignant T cells in patients with CLL is poorly understood. In the present study, using FCM, we evaluated the modulation of T lymphocytes in patients with CLL receiving venetoclax front-line therapy. Examining the percentage of BCL-2-positive lymphocytes between untreated patients and those in CR in T cells that exhibited intra-individual variations, we documented that venetoclax significantly shaped the T cell profile, inducing an in vivo decrease in BCL-2 expression in CD4^+^ and CD8^+^ T cells. This observation supports the hypothesis that the expression of BCL-2 in T lymphocytes is inhibited by venetoclax and is consistent with the report by Mathew et al. that venetoclax reduces the immunosuppressive environment by decreasing the percentage of Tregs and PD-1^+^Tim-3^+^CD8^+^ T cells [64]. These findings indicate that venetoclax can serve as a positive factor for reversing immune suppression and provide a strong theoretical basis for combined immunotherapy with venetoclax in CLL and other cancers.

Our study identified a new marker BCL-2, which can distinguish patients with CLL having T cell defects. However, our research might require further refinement on how BCL-2 overexpression affects T cell dysfunction in patients with CLL, which will be explored in future biological experiments.

## Conclusions

In summary, our study revealed the roles of BCL-2 expression in the T cells of patients with CLL. BCL-2 promoted immunosuppression by enhancing Treg differentiation and CTL cell exhaustion. Our findings identify a new strategy for using the BCL-2 inhibitor, venetoclax, for CLL treatment and provide a new paradigm for restoring T cell function.

## Supplementary Information

**Additional File 1: Supplementary Fig. S1.** Representative flow cytometry images of BCL-2 expression in CD3^+^ (a), CD4^+^ (b), and CD8^+^ (c) T cells between patients with CLL and normal controls.

**Additional File 2: Supplementary Fig. S2.** Bubble plot of PBMC markers in scRNA-seq.

**Additional File 3: Supplementary Fig. S3.** (a) The violin plots represent the data distribution of three genes *PRF1*, *GZMB*, and *FASLG* in *BCL2-*positive and -negative T cells. In the box plot, the boxes hold 50% of the data, with an equal number of data points above and below the median deviation (full gray line). Outliers beyond this range are indicated with circular makers. (b) Pearson’s correlation analysis was performed to analyze the correlation between *BCL2* expression in T cells and two gene sets indicated in the figure. (c) The violin plots represent the data distribution of the normalized enrichment score of *BCL2*-positive and -negative T cells of the gene sets indicated in the figure. In the box plot, the boxes hold 50% of the data, with equal number of data points above and below the median deviation (full gray line). Outliers beyond this range are indicated with circular makers.

**Additional File 4: Supplementary Fig. S4.** Percentage of Tn, Tem, Tcm, and Teff without BCL-2 subgrouping in CD4^+^ (a) and CD8^+^ (b) T cells in 70 patients with CLL.

**Additional File 5: Table S1.** Key Resources

## Data Availability

The datasets used and/or analyzed during the current study are available from the corresponding author on reasonable request.

## Abbreviations

CLL: Chronic lymphocytic leukemia
BCL-2: B-cell lymphoma 2
scRNA-seq: single-cell RNA sequencing
Treg: regulatory T cell
CTL: cytotoxic T lymphocyte
TME: tumor microenvironment
AML: acute myeloid leukemia
FCM: flow cytometry
CR: complete remission
PBMCs: peripheral blood mononuclear cells
PBS: phosphate-buffered saline
PD-1: programmed cell death protein 1
TIM-3: T cell immunoglobulin domain and mucin domain-3
Th: T helper
Ig: immunoglobulin
SSC: side scatter
FSC: forward scatter
IFN-γ: interferon-gamma
IL: interleukin
TGF-β: transforming growth factor beta
β2-MG: β2-microglobulin
MFI: Mean fluorescence intensity
NC: normal control
CLL-IPI: CLL- International Prognostic Index
UMAP: Uniform Manifold Approximation and Projection
DEGs: differentially expression genes
GSEA: Gene set enrichment analysis
GSVA: gene set variation analysis
TFs: transcriptional factors
TFT: transcription factor target
MSigDB: Molecular Signatures Database
Tfh: follicular helper T cell
Teff: effector T cells
Tem: effector memory T cells
Tcm: central memory T cells
TCR: T cell receptor
