# Supplementary figures and images for: BCL-2 expression promotes immunosuppression in chronic lymphocytic leukemia by enhancing regulatory T cell differentiation and cytotoxic T cell exhaustion
Source: Mol Cancer. 2022 Feb 22;21:59. doi: 10.1186/s12943-022-01516-w (PMC8862474; doi:10.1186/s12943-022-01516-w)

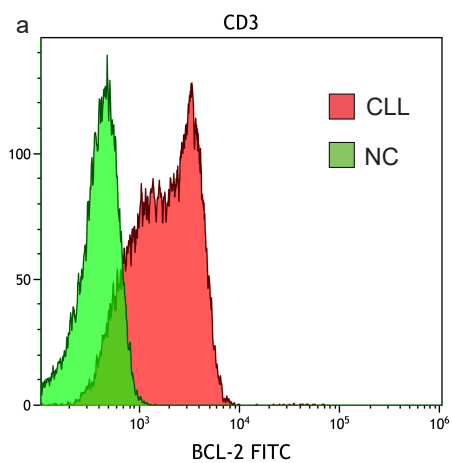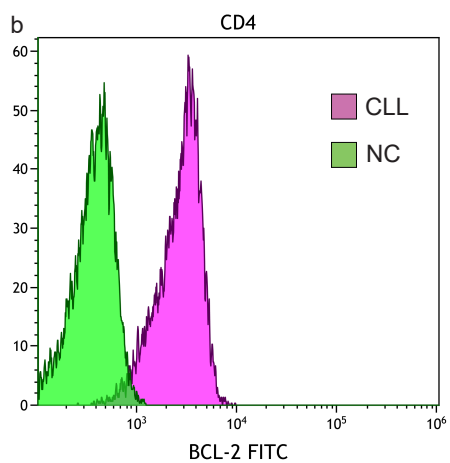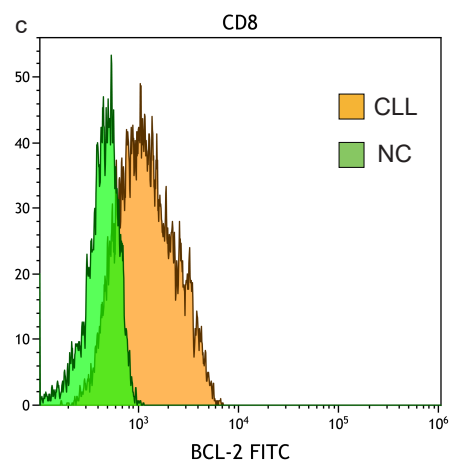

Supplement: Supplementary file 1 — Additional File 1: Supplementary Fig. S1. Representative flow cytometry images of BCL-2 expression in CD3+ (a), CD4+ (b), and CD8+ (c) T cells between patients with CLL and normal controls. [file 12943_2022_1516_MOESM1_ESM.pdf]

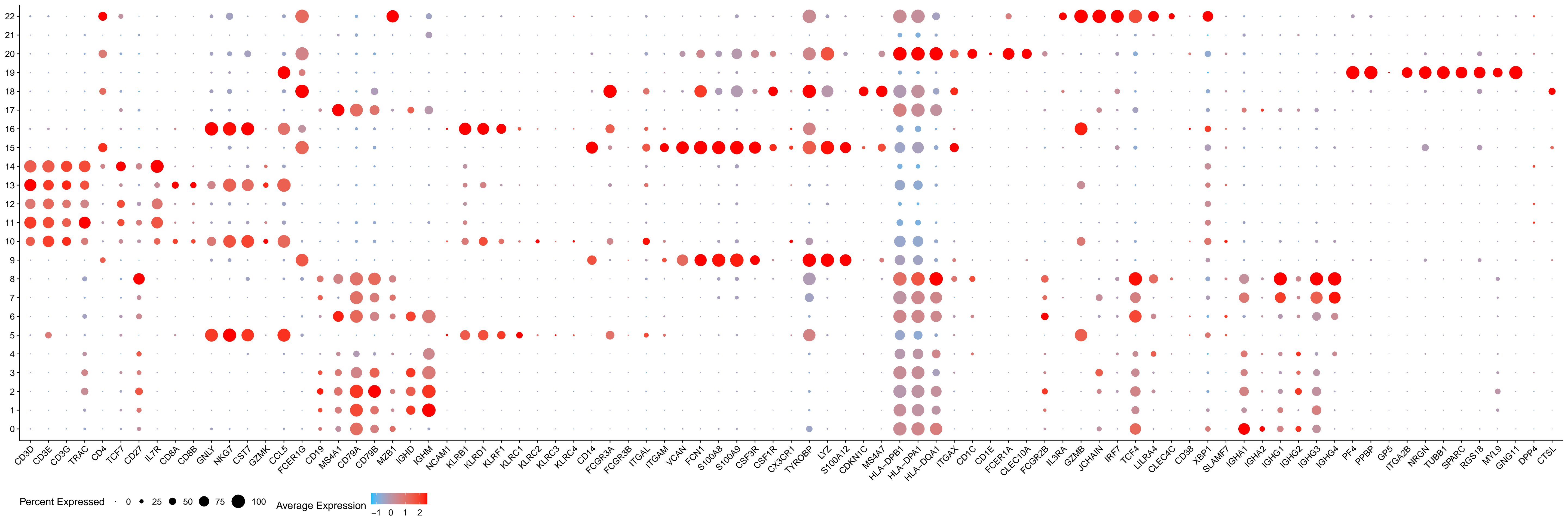

Supplement: Supplementary file 2 — Additional File 2: Supplementary Fig. S2. Bubble plot of PBMC markers in scRNA-seq. [file 12943_2022_1516_MOESM2_ESM.pdf]

**a**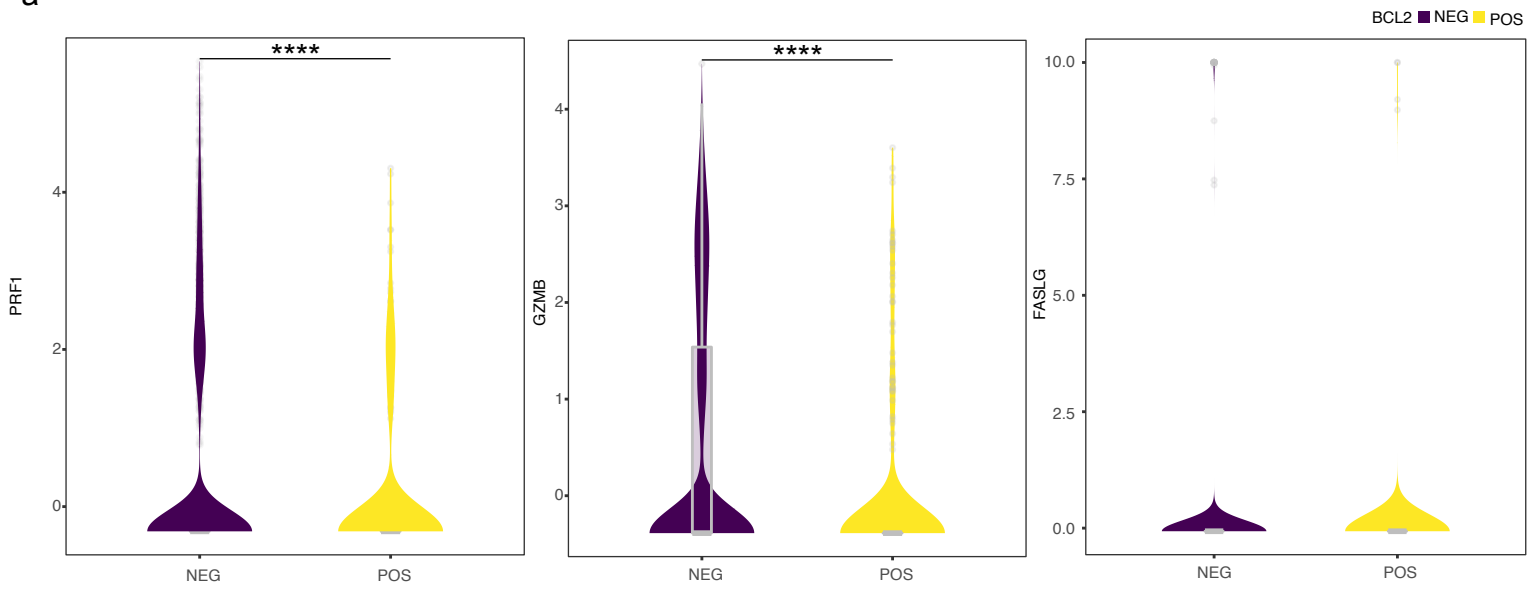**b**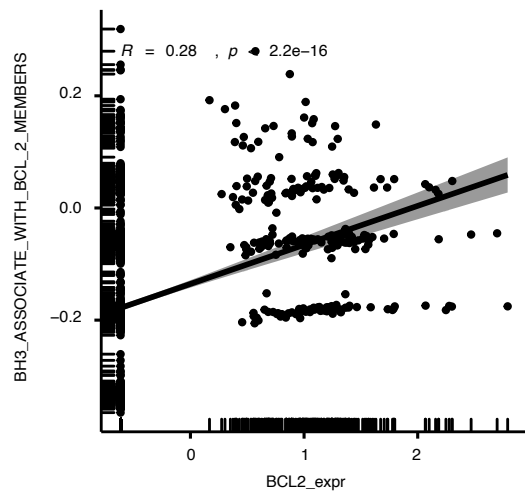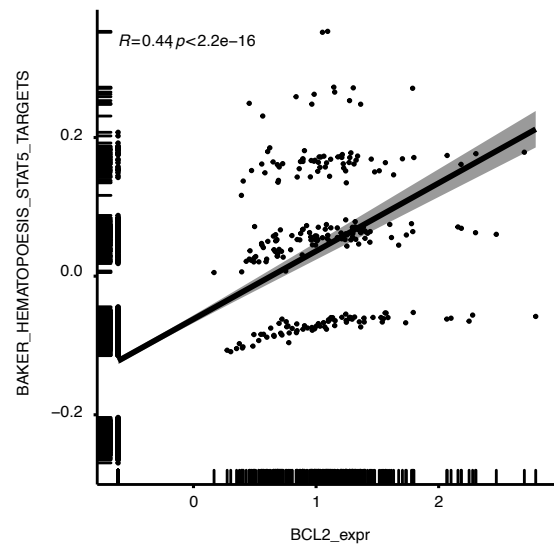**c**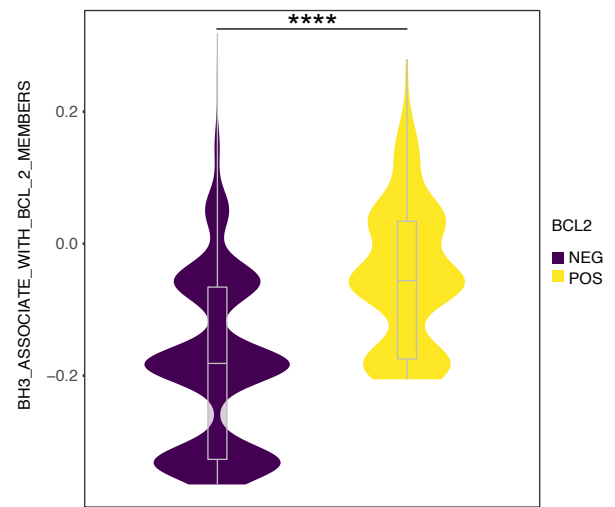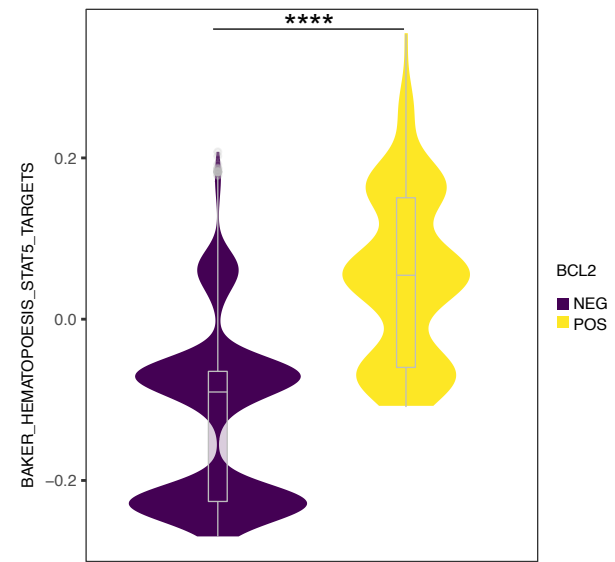

Supplement: Supplementary file 3 — Additional File 3: Supplementary Fig. S3. (a) The violin plots represent the data distribution of three genes PRF1, GZMB, and FASLG in BCL2-positive and -negative T cells. In the box plot, the boxes hold 50% of the data, with an equal number of data points above and below the median deviation (full gray line). Outliers beyond this range are indicated with circular makers. (b) Pearson’s correlation analysis was performed to analyze the correlation between BCL2 expression in T cells and two gene sets indicated in the figure. (c) The violin plots represent the data distribution of the normalized enrichment score of BCL2-positive and -negative T cells of the gene sets indicated in the figure. In the box plot, the boxes hold 50% of the data, with equal number of data points above and below the median deviation (full gray line). Outliers beyond this range are indicated with circular makers. [file 12943_2022_1516_MOESM3_ESM.pdf]

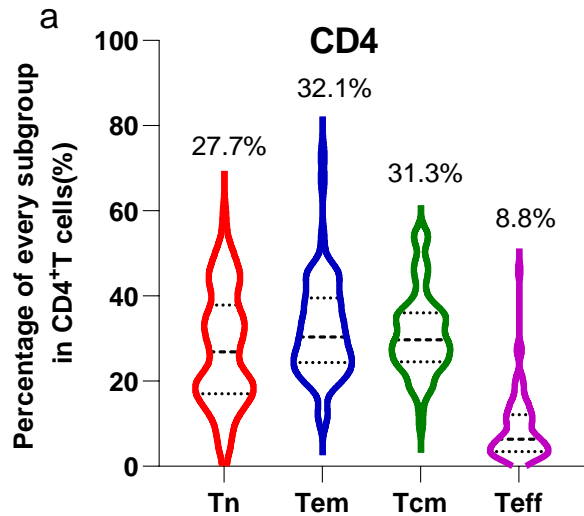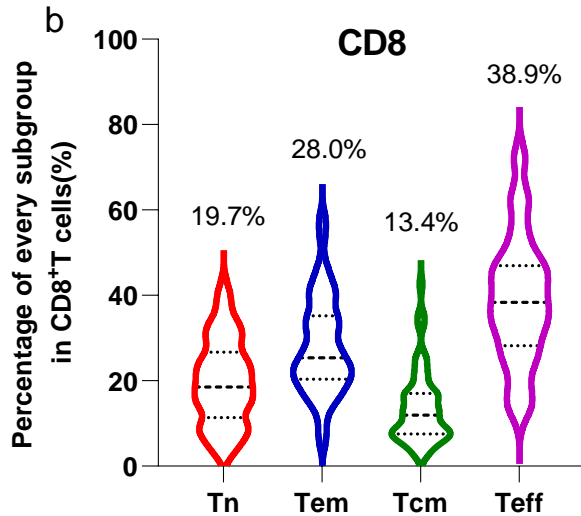

Supplement: Supplementary file 4 — Additional File 4: Supplementary Fig. S4. Percentage of Tn, Tem, Tcm, and Teff without BCL-2 subgrouping in CD4+ (a) and CD8+ (b) T cells in 70 patients with CLL. [file 12943_2022_1516_MOESM4_ESM.pdf]
