# Supplementary material for: BCL-2 expression promotes immunosuppression in chronic lymphocytic leukemia by enhancing regulatory T cell differentiation and cytotoxic T cell exhaustion
Source: Mol Cancer. 2022 Feb 22;21:59. doi: 10.1186/s12943-022-01516-w (PMC8862474; doi:10.1186/s12943-022-01516-w)
Supplement: Supplementary file 5 — Additional File 5: Table S1. Key Resources [file 12943_2022_1516_MOESM5_ESM.docx]

| REAGENT or RESOURCE | SOURCE | IDENTIFIER |
| --- | --- | --- |
| Antibodies |  | CLONE |
| Monoclonal Mouse Anti-Human BCL2 Oncoprotein/FITC | DAKO | 124 |
| PE anti-human IFN-γ | BioLegend | 4S.B3 |
| Anti-human Foxp3 PE | Invitrogen | PCH101 |
| PE Mouse Anti-Human Granzyme B | BD Biosciences | GB11 |
| PE anti-human LAP (TGF-β1) | BioLegend | TW4-2F8 |
| PE Mouse Anti-Human CD4 | BD Biosciences | SK3 |
| PE Mouse Anti-Human CD8 | BD Biosciences | SK1 |
| CD3-ECD | Beckman Coulter | UCHT1 |
| CD8-ECD | Beckman Coulter | SFCI21Thy2D3 |
| CD19Percp-Cy5.5 | BD Biosciences | SJ25C1 |
| CD25-PC5 | Beckman Coulter | NA |
| CD4 PE-CY7 | BD Biosciences | SK3 |
| PE-Cy7 anti-human IL-22 | BioLegend | 2G12A41 |
| PE-Cy™7 Mouse Anti-Human CD45RO | BD Biosciences | UCHL1 |
| APC anti-human Perforin | BioLegend | dG9 |
| APC anti-human IL-10 | BioLegend | JES3-9D7 |
| APC anti-human CD366 (Tim-3) | BioLegend | F38-2E2 |
| APC anti-human CD185 (CXCR5) | BioLegend | J252D4 |
| Human IL-12/IL-35 p35 Alexa Fluor® 700-conjugated | R&D Systems | NA |
| APC/Cyanine7 anti-human CD62L | BioLegend | DREG-56 |
| Brilliant Violet 421™ anti-human CD279 (PD-1) | BioLegend | EH12.2H7 |
| Brilliant Violet 421™ anti-human CD8 | BioLegend | SK1 |
| Brilliant Violet 421™ anti-human CD45RA | BD Biosciences | HI100 |
| Brilliant Violet 510 anti-human IL-17A | BioLegend | BL168 |
| Brilliant Violet 510 anti-human IL-4 | BioLegend | MP4-25D2 |
| BV510 Mouse Anti-Human CD3 | BD Biosciences | UCHT1 |
| Reagents |  | Cat. |
| eBioscience™ Human Regulatory T Cell Staining Kit | Invitrogen | 88-8999-40 |
| Cell Activation Cocktail (with Brefeldin A) | BioLegend | 423303 |
| Stain Buffer (with 2% FBS and 0.09% Sodium azide) | BD Pharmingen | 554656 |
| Phosphate Buffered Saline (1×) | HyClone | SH30256.01 |
| Lymphocyte Separation Medium | TBD | LTS1077 |
| RPMI 1640 medium | Gibco | 11875093 |
| Fetal Bovine Serum | Gibco | 3160802 |
| Penicillin-Streptomycin | Gibco | 15140122 |
| IntraStain Kit | DAKO | K2311 |
